# [Corrigendum] Effects of miR-340 overexpression and knockdown on the proliferation and metastasis of NSCLC cell lines

**DOI:** 10.3892/ijmm.2024.5433

**Published:** 2024-09-30

**Authors:** Xidan Zhu, Gang Tian, Jing Quan, Peng He, Jinbo Liu

Int J Mol Med 44: 643-651, 2019; DOI: 10.3892/ijmm.2019.4213

Following the publication of the above article, an interested reader drew to the authors' attention that, with the 'Adjacent' row (top row) of immunohistochemical images shown in Fig. 2 on p. 646, the fourth and fifth panels along (the 'RAB11A' and 'RAB9A' data panels) contained an overlapping section of data, such that data which were intended to show the results from differently performed experiments had apparently been derived from the same original source.

After consulting their original data, the authors were able to determine that the duplication of these panels had inadvertently occurred during the process of compiling Fig. 2. The revised version of Fig. 2, featuring the correct data for the 'Adjacent/RAB9A' experiment, is shown below. The authors confirm that the error associated with this figure did not have any significant impact on either the results or the conclusions reported in this study, and are grateful to the Editor of *International Journal of Molecular Medicine* for allowing them the opportunity to publish this Corrigendum. Furthermore, they apologize to the readership of the Journal for any inconvenience caused.

## Figures and Tables

**Figure 2:**
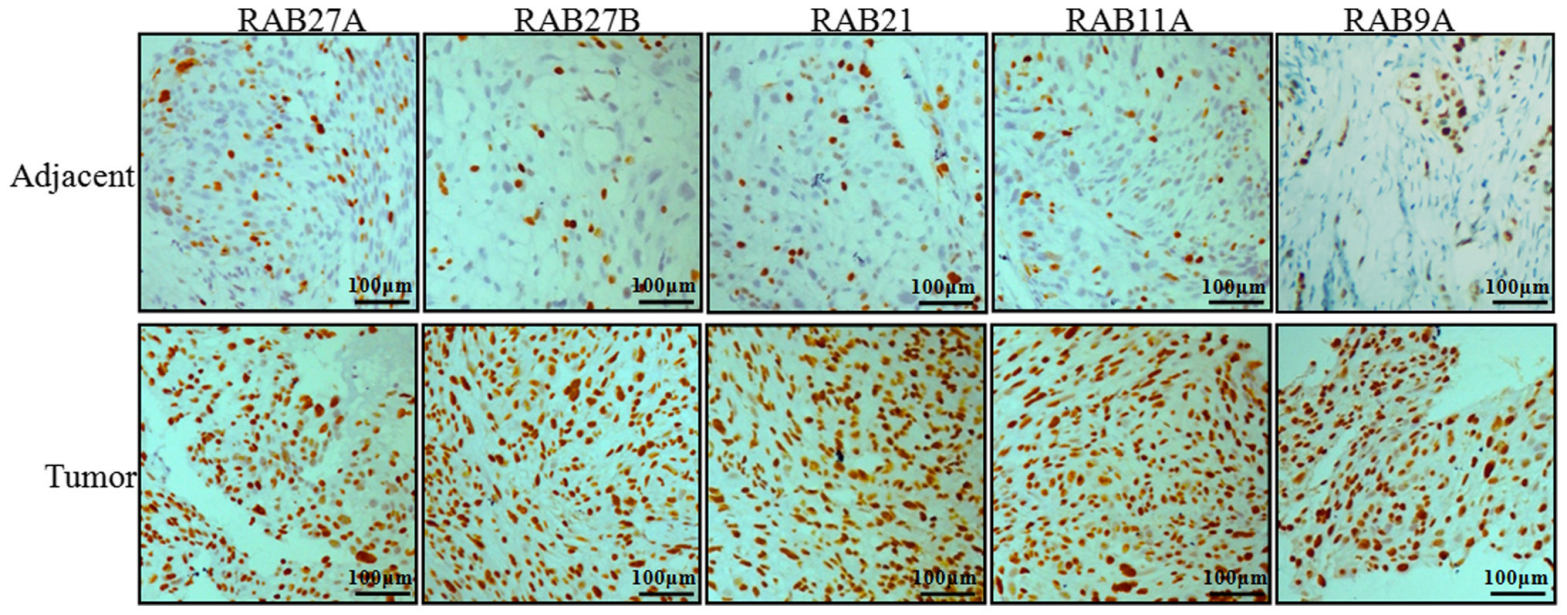
Immunohistochemical analysis of RAB family proteins in NSCLC tissues. The expression of the RAB family of proteins, including RAB27A, RAB27B, RAB21, RAB11A and RAB9A in tumor and adjacent tissues of patients with NSCLC was analyzed by immunohistochemistry and observed under an inverted microscope. NSCLC, non-small cell lung cancer.

